# The Co-Creation and Implementation of a Protocol for the Prevention of Gender Violence in a Non-University Adult Educational Center

**DOI:** 10.3390/bs15040406

**Published:** 2025-03-23

**Authors:** Alba Crespo-López, Rosa Valls-Carol, Elisenda Giner-Gota

**Affiliations:** 1 Department of Theory and History of Education, University of Barcelona, 08035 Barcelona, Spain; albacrespo@ub.edu; 2 Department of Linguistic, Scientific and Mathematical Education, University of Barcelona, 08035 Barcelona, Spain; eginer@ub.edu

**Keywords:** gender violence, protocol, co-creation, violent behavior, the “other women”

## Abstract

The scientific literature presents evidence of the processes involved in creating and developing protocols aimed at maintaining a safe educational and work environment that prevents gender violence in universities, which has shown negative neuropsychological health effects at the individual and community levels. However, to date, there have been no scientific publications analyzing the characteristics of protocols that have been successful in preventing gender violence at non-university educational centers. To address this gap, and in the context of advancing the SDG 5 challenge “Gender equality”, a qualitative case study was conducted to analyze the social and behavioral aspects involved in the creation and implementation of a protocol for preventing and addressing gender violence at an urban adult school in Spain. The findings indicate that the co-creation and implementation of the protocol, involving women participants without higher education degrees, researchers, and educators from the school, contributed to better upstander behavior through support for victims from the very beginning, contributing to the creation of a space free from violent behavior that offers protection from its harmful psychological consequences.

## 1. Introduction

In the face of the numerous cases of gender violence and the serious consequences it has on victims, their environments, and communities (WHO, 2024), the development of measures to prevent it have begun to shape specific policies. Regarding the educational context, the creation and implementation of protocols to address gender violence have been an issue that has received little attention, especially in the non-university adult education context, which predominantly includes adult women without academic qualifications who suffer the greatest discrimination as a result (Ruiz-Eugenio et al., 2024). The social and behavioral characteristics and processes that have shaped the development of these protocols are therefore of relevance for overcoming gender violence and preventing its psychological consequences, which is an urgent global health problem that needs to be addressed (Lausi et al., 2024).

In 1995, an international definition of gender violence was established during the United Nations Fourth World Conference on Women in Beijing (United Nations, 1995). This definition recognizes the physical, emotional, psychological, and economic dimensions of gender violence and that it occurs in all contexts, assigning responsibility to governments and organizations to create and implement policies to prevent and eradicate it (United Nations, 1995). Nonetheless, prior to this declaration, reflections, dialogues, and research on gender violence were already taking place, leading to measures aimed at eliminating it (Cook et al., 2018). The United Nations has defined gender-based violence as violence against women specifically because they are women, or as violence that disproportionately affects women (United Nations, 1993).

### 1.1. Scientific Evidence of Social Impact Preventing Gender Violence

Studies in a wide range of contexts have shown that, in order to prevent mental health problems such as suicidal behaviors, it is essential to improve cohesion between people and eradicate abuse and violence (Sher, 2024). Research has shown that engaging in supportive groups improves financial, health, and legal issues, as emotional support seems to be the most beneficial for the psychological and physical health of victims (Erčulj & Pavšič Mrevlje, 2023). In this regard, previous studies have demonstrated the positive impacts of social support on health, and have shown that perceived social support significantly and negatively predicts mental health problems (Gao et al., 2024). Indeed, an important milestone in the success of actions against violence is ensuring support for victims by empowering bystanders to become upstanders (Puigvert et al., 2022), which means transforming individuals who witness violent acts into individuals who act against or prevent them (Banyard et al., 2007).

Nonetheless, bystanders do not always intervene and become upstanders, primarily due to the risk of reprisals and negative consequences (Puigvert et al., 2021). Some upstanders even become victims of violence themselves for supporting direct victims (Cañaveras et al., 2024). In 2020, this form of violence against those who protect victims was legally recognized and legislated under the term second-order violence (SOV) by the Catalan Parliament through a unanimous vote, asserting itself as a fundamental factor to be incorporated into all measures aimed at preventing gender violence in any context (Vidu et al., 2022). Subsequently, it was conceptualized with the term isolating gender violence (IGV) (Aubert & Flecha, 2021). Research has shown that IGV results in mental health damage, not only to those who directly receive attacks for protecting the victims but also to their environments, including their family members, who also suffer adverse health consequences. Overcoming IGV is therefore an urgent health problem that needs to be addressed to eliminate gender violence (Flecha et al., 2024), as victim support and networking have been essential to victims breaking their silence and becoming survivors (Cañaveras et al., 2024).

The involvement of the community is crucial for eradicating violence. In the INCLUDE-ED project (INCLUD-ED Project, 2006–2011; Flecha, 2015), funded by the European Commission, researchers analyzed educational strategies that contribute to overcoming inequalities and fostering social cohesion, demonstrating that the involvement of the community in creating a consensual norm of coexistence free from violent behavior, through egalitarian dialogue, has an impact on preventing violence (Gatt et al., 2011). This consensus of the norm was conducted within the Dialogic Model of Prevention and Resolution of Conflicts (DMPRC) framework, which is a Successful Educational Action (SEA) that promotes a form of socialization that associates attractiveness with non-violent actions by reinforcing upstander behavior while rejecting violence (Villarejo-Carballido et al., 2019).

### 1.2. The “Other Women” in the Process of Creating and Implementing Actions for the Prevention of and Attention to Gender Violence

Throughout history, women who have not had access to academic education have been relegated from spaces of debate and decision making, which has silenced their voices (Beck-Gernsheim et al., 2003). Lidia Puigvert, together with women participants in adult education, coined the term “other women” to refer to adult women without an academic education (Puigvert, 2001). The inclusion of the “other women” has contributed to successful actions against violence in various contexts, including adult education institutions, their children’s schools (Ruiz-Eugenio et al., 2023), and the community (Valls Carol, 2014). Their inclusion in the co-creation processes of the actions conducted in health centers, such as dialogic gatherings, is having a positive impact on their mental health and well-being (Padrós-Cuxart et al., 2024). Previous studies have shown that schools implementing actions based on egalitarian dialogue, including the “other women”, have developed effective mechanisms for identifying and preventing gender violence (Oliver et al., 2009).

### 1.3. Protocols for the Prevention of and Action Against Gender Violence at Universities

No scientific articles were found in the existing literature regarding protocols for the prevention of and intervention in gender violence in non-university educational centers for adults. However, in the university context, pioneering research in Spain has made progress on this issue through providing scientific contributions on protocols against gender violence in universities. In September 1991, the Community of Researchers on Excellence for All (CREA) was founded, with committed actions to eradicate gender violence based on scientific evidence. Its women’s group (Safo) became the only university group to break the silence on gender violence within Spanish universities in 2003 (Joanpere et al., 2022). In 2003, CREA designed the first research project on violence against women in Spanish universities, which later became an R + D + i project (2005–2008) (Valls et al., 2016). This research advocated for policies and actions to be based on zero tolerance towards any type of gender violence, as well as for intervention, support, and solidarity with the victims and the people who support them, leading to measures that required universities to have equality commissions and protocols against gender violence, which was reflected in 2007 in the Spanish Law (Government of Spain, 2007). Since then, many university protocols for prevention and intervention in cases of gender violence have been developed from Equality Units and Commissions in Spain. Subsequently, in both the U.S. and Spain, MeToo University—a university movement that originated in 2013—made considerable strides in academia, including the promotion of all universities to implement protocols for action in cases of sexual harassment and gender violence (Joanpere et al., 2022).

Regarding the international state of the art, gender violence at universities is now considered a global public health problem, and protocols to prevent it are now established in diverse countries (Berkeley PATH to Care Center, 2020), as the feminist movement within universities brought about changes in higher education institutions and introduced protocols to address discrimination and gender violence. However, significant challenges to advancing and implementing gender policies have been identified. Evidence points to the importance of establishing protocols to prevent gender violence in universities and its mental health consequences for students (Assari & Moghani Lankarani, 2018); however, these protocols are sometimes ineffective and exclude many women’s voices, and they often focus only on offering victims mechanisms to formally complain (Masinire & Sanchez-Cruz, 2023).

According to a review of the existing literature, there is no known published protocol on the prevention of gender violence at non-university adult schools. Despite the increasing measures that have been implemented to prevent gender violence at universities, there is a lack of studies on protocols for the prevention of and response to gender violence at non-university educational institutions for adult people. To fill this gap in the literature, a qualitative case study was developed on the only known case. Within the framework of the R + D + i research project ALL WOMEN “The empowerment of all women through adult education for a sustainable development” (PID2020-113137RA-I00), funded by the State Research Agency of the Ministry of Science, Innovation and Universities of Spain, the process of the co-creation of the protocol for the prevention of gender violence among “other women”, researchers, and educators and its implementation in an adult school were analyzed. The present qualitative case study aims to fill the gap in the literature by answering the following two research questions:

**RQ1:**  *How has the protocol for dealing with gender violence at an adult school been created?*

**RQ2:**  *How is this protocol implemented?*

## 2. Materials and Methods

In this study, we analyzed participants’ perceptions regarding the creation and implementation processes and characteristics of a protocol for the prevention of gender violence at a non-university adult educational center. This qualitative case study was conducted through the communicative methodology, including participants in the whole research process (Puigvert et al., 2012). The participants—who, in this case, were women without academic degrees who participated at the adult school, educators at the school, and collaborating researchers—contributed their perceptions through their stories and in a discussion group in egalitarian dialogue with the researcher of the team in the creation of knowledge. In addition, the validation of the results was discussed with the women participants on an advisory board once the information had been analyzed and the results defined.

### 2.1. The Study Context and Participants

#### 2.1.1. La Verneda-Sant Martí Adult School

La Verneda-Sant Martí Adult School, created in 1978 and located in what was then a low-income neighborhood in Barcelona, serves as an international reference due to its trajectory and its contributions to the transformative movement in democratic education (Sánchez-Aroca, 1999). This adult school implements Successful Educational Actions (SEAs) that improve learning for diverse people while contributing to the social cohesion of the community, and it was the first school constituted as a Learning Community (Aubert et al., 2016). This school emerged in the context of the neighborhood movements that arose at the end of the Franco dictatorship in Spain, driven by community participation, which led to the establishment of many adult education schools to address the literacy needs of the population (Gómez Cuevas & Valls, 2022). Based on the principles of dialogical learning (Flecha, 1997/2000), this school carries out educational actions that the community identifies as necessary in a democratic and egalitarian manner in co-creation with all participants and based on the actions that science has evidenced to have an impact on improving learning and social cohesion. In this framework, a protocol for the prevention of gender violence at this non-university adult educational center was implemented (Soler-Gallart & Joanpere, 2024).

However, prevention and action against gender violence at this adult school did not begin with the establishment of its written protocol in the early 2000s. From its origin in 1978, the school was intended to be a space free from all violence. From its beginnings, participants, collaborators, and educators at the adult school have been aware of the existence of gender violence, what gender violence means, and that it can occur in all social contexts. The educational project of this school, which offers activities from Monday to Sunday from 9 am to 10 pm, with more than 2000 enrollments each academic year and over 100 collaborators, has addressed gender violence from the very beginning.

Therefore, all people involved in this adult school are adamant that gender violence must be identified, prevented, and acted against, with the school promoting egalitarian interactions, facilitating discussions on how to prevent violence, and conducting actions in specific cases. This protocol was created according to all of the school’s previous knowledge and experience from dialogues and actions, in this respect. When this protocol was established in writing, it was reviewed by the women’s group of this adult school, as well as by the school’s Anti-Harassment Committee, to ensure that the content of the protocol was correct.

#### 2.1.2. The Protocol for the Prevention of Gender Violence at La Verneda-Sant Martí Adult School

Every academic year, the protocol is presented in this school in the languages that represent the school’s participants which currently include nine different languages (Spanish, Catalan, English, French, Chinese, Russian, Ukrainian, Arabic, and Urdu), so that everyone involved in the school are aware of how it works. The protocol presents the consensus on the norms of coexistence, the positioning of zero violence, and the mechanisms of denunciation.

The norm of coexistence of La Verneda-Sant Martí Adult School is based on human rights within the Dialogic Model of Conflict Prevention and Resolution (DMCPR) framework and from an educational perspective, and follows the guidelines of the IV World Conference on Women (United Nations, 1995), the Istanbul Convention (Council of Europe, 2014), and Law 17/2020, of 22 December, on the right of women to eradicate gender violence, as no attitude or comment that violates the rights of any person is allowed in the school. In addition, the school has an Anti-Harassment Committee to ensure that it remains a space free from any type of harassment.

The protocol’s approach to addressing human rights violations, harassment, or gender violence includes providing information on how to handle cases of violence and involves the Anti-Harassment Committee, composed of participants, collaborators, and individuals from diverse cultures and age groups, who consistently support victims. This protocol includes information for everyone that is linked to the school: “*Come to school to learn and enjoy, don’t worry*”; “*No man at school can make you uncomfortable: neither inside nor outside the school*”; “*At school you won’t have to meet someone who harasses you*”; “*Feel free, you decide: Yes or no to a photo, to being accompanied, to that joke, to that proximity…*”; “*Be brave, stand in solidarity with those who defend women victims*”; “*If you experience an uncomfortable situation, report it*”; “*If you live in an uncomfortable situation, report it*”; “*Be brave, stand in solidarity with those who defend women victims*”; “*If you live in an uncomfortable situation, report it*”; “*A school free of violence needs courageous men and women who take a stand against sexist, chauvinist and harassing attitudes, who always stand in solidarity with the woman victim and her support network, who always respect and accompany the woman in her free choice, without coercion, without pressure*”.

The protocol also outlines the mechanisms for denouncing any violent act: “*there is a specific Committee that will analyze cases that have not been solved or are in doubt. This Committee proposes the intervention to the coordination of the entity, always taking a position in favor of the victims. As a general rule, no one who is not committed to changing sexist behavior is allowed to remain in the organization*”. The school offers three different reporting methods—in person, by phone, or by mail—all of them confidential. In addition, civil reporting mechanisms for cases of gender violence are provided so that anyone who observes violent behavior can report it inside and outside the school.

### 2.2. Participants

In this study, we gathered the perceptions of individuals involved in the creation and implementation of this protocol regarding its development process and characteristics.

Thus, this qualitative case study included the participation of six women with different profiles through interviews, life stories, and a focus group, as shown in Table 1. The selection criteria for the profiles were as follows: involvement in La Verneda-Sant Martí Adult School and participation in the design, implementation, or dissemination of the analyzed protocol to engage in dialogues revealing key information about its processes and characteristics.

To collect the data, three in-depth interviews were conducted in person, one with each of the three educators of La Verneda-Sant Martí Adult School; three in-person life stories from non-academic women participants at the adult school; and an online focus group with two top researchers on gender violence prevention and one non-academic woman participant at the adult school.

All data collection techniques were used by members of the research team, who explained the study’s purpose and the participants’ role in it, including how their data would be treated. All participants received and signed an informed consent form, and all of them engaged in open conversations with the researcher, who directed these dialogues towards the research questions, which concerned the creation and implementation of this protocol.

The profiles of the participants when displaying quotes expressing their contributions are coded using the following identifiers to maintain their anonymity: I: interview; LS: life story; FG: focus group; E: educator; Pa: participant; R: researcher; W: woman.

### 2.3. Analysis Categories

All the information collected was recorded and transcribed by the research team, who then independently analyzed the information to select quotes that corresponded to the research questions and that therefore provided information about the participants’ perceptions on the processes involved in the development of the study protocol and its characteristics. Following that, the research team, in dialogue with the participants, reached a consensus on the following categories of analysis: regarding RQ1 (“How has the protocol for dealing with gender violence in an adult school been created?”), the categories were as follows: *(1) commitment as a starting point* and *(2) the co-creation process*; regarding RQ2 (“How is this protocol implemented?”), the categories were as follows: *(3) the inclusion of the “other women” in dissemination*, *(4) the inclusion of the “other women” in implementation*, and *(5) the promotion of upstander behavior*.

## 3. Results

The analysis of the results is presented in two sections, corresponding to the two research questions. The first section focuses on the commitment to gender violence prevention and being in favor of victims since the creation of the school, in addition to the co-creation of the protocol. The second section addresses the inclusion of “other women” in the dissemination and implementation of the protocol, along with the promotion of upstander behavior.

### 3.1. RQ1: How Has the Protocol for Dealing with Gender Violence in an Adult School Been Created?

#### 3.1.1. Commitment to Take a Stand Against Gender Violence and Being in Favor of Victims Since the Creation of the School

Educator 2, one of the educators at La Verneda-Sant Martí Adult School, who has been collaborating there for over 25 years and is involved in the movement of participants in adult education and with “other women”, explains that, since the school’s creation in 1978, participants have adopted a clearly feminist approach, committed to addressing the educational needs of all women with solidarity and support throughout their processes. She clarifies that Dr. Ramón Flecha, the school’s founder, played a pivotal role in establishing and promoting this commitment. Since its inception, this adult education school has addressed cases of gender violence and other issues for which these women may need support. In 1978, the school collaborated in the creation of the first family planning center after the dictatorship ended, where women could seek advice on sexual and reproductive health. Within this framework of the school’s collaboration with the family planning center, a manual for the early detection of gender violence was created. The educator emphasizes that this support has always been provided with respect for women’s freedom of choice, supporting them in cases in which such attention was both requested and consented to. Therefore, from the outset, if a woman faced issues related to gender violence, then she was supported if she so wished. “Flecha, the founder of the school, greatly promoted this approach, which has been present from the very start. He and the female doctor from the family planning center created a manual for the early detection of gender violence (I_W_E2)”.

Researcher 2, who participated in the discussion group, is a recognized academic in the field of dialogic feminism, and has been a long-time collaborator with the school, acting on the implementation of this protocol, indicated that, from the school’s beginning, a manual was created to identify cases of aggression and gender violence at an early stage, long before the current protocol was written: “In fact, they did it at that time, for health prevention and identifying cases of aggression, and it was done right from the start of the school (FG_W_R2)”.

Educator 2 highlights that the creation of the protocol was one of the proactive measures taken to prevent potential sexual harassment within the school, where hundreds of people participate every day. She emphasizes that at a certain point, the need for a formal protocol against sexual harassment became evident as a logical step to ensure that the school was a safe space, in line with what had already been executed since 1978:

At a given moment, a protocol against sexual harassment had to be drawn up, why? Well, because it is considered that in an organization where there are thousands of people living together, there can logically be a risk of sexual harassment. And so, since 1978, we had already tried to make it a very safe place for women, and it had been.

(I_W_E2)

In relation to the Spanish legal context, it was in 2004 (Government of Spain, 2004) and 2007 (Government of Spain, 2007) when legislation came into force that regulated the establishment of protocols for the prevention of gender violence in different institutions. The protocol of La Verneda-Sant Martí Adult School predates this legislation. Researcher 2, who studies feminism and is a collaborator of the school, specifies that the school had frameworks and measures in place on how to identify, for example, whether a woman arrived at the school with physical signs of having suffered aggression. This researcher explains that the protocol was the formal systematization of these existing measures. By creating the protocol, they organized these measures into a structured, step-by-step process:

I already had it because from the beginning of the school there were frameworks on how to identify if a woman could come with situations of physical aggression on her body, for example how to identify them. The only thing we did with the protocol was to systematize something that already existed in the school. We systematized it, we simply put a bit of ‘step one, step two, step three…’.

(FG_W_R2)

Educator 2 comments that the main objective of this protocol was to ensure safety for all women attending the school, as well as to ensure that all women can disclose any dangerous situations:

So, the main objective of the Protocol is that every woman can feel safe in school and that anything that she considers to be an attack on her, on her dignity as a woman can be explained and shared.

(I_W_E2)

#### 3.1.2. Co-Creation of the Protocol

When the participants of this adult school decided to formalize the measures they had already been implementing to prevent and address gender violence in their institution by documenting them in a protocol, they found no evidence of any previous creation or implementation of such a protocol at any non-university adult education center. Educator 2 stated that the creation of this protocol was based on existing scientific evidence-based actions to create spaces free from gender violence, as well as the guidelines provided by international organizations such as the UN (United Nations) and WHO (World Health Organization). In line with the democratic and dialogic model of this school, and building on all the school’s prior knowledge and experience, a committee was created consisting of non-academic female participants, educators, and researchers on gender violence who had been collaborating with the school since its inception to develop the protocol:

The protocol is made according to international guidelines. We looked at what existed and how to carry it out. It was considered appropriate to create a Committee that would have represented participants (non-academic women), researchers in the field of gender violence, collaborators, etc.

(I_W_E2)

Researcher 1, who is also a woman who has been recognized for her research on gender violence and who has been a collaborator of this school since its very beginning, explained how, in the meetings with non-academic women in which the creation of the protocol was being worked on, they, as researchers, brought scientific evidence to the debates about what contributed to creating spaces free of gender violence. In the egalitarian dialogue between the researchers, educators, and non-academic women, based on this scientific evidence and the school’s prior experience, the protocol was agreed upon. This researcher emphasized that they contributed scientific evidence to the debate and not the imposition of what had to be accomplished, as the protocol was the result of dialogue between them all: “We bring to the protocol a bit of scientific evidence. It is based on all the research that has been done on what works on a scientific basis (FG_W_R1)”.

In the focus group, Researcher 2 added a key element to the analysis. First, she emphasized what Researcher 1 had already mentioned: that the school has always aligned itself with scientific evidence. Second, she highlighted the school’s ongoing concern for the neighborhood, given that it is part of the local community. These two factors combined meant that when gender violence began to gain some social recognition, the school, being connected both with people in the scientific field and the community, recognized the need to be a pioneer in developing its own protocol. This happened at a time when such protocols were becoming mandatory for all institutions and workplaces, but few had them. Researcher 2 emphasized that this adult school was a pioneer in adopting this measure:

Well, I would add two things. One is that the school, as this school has always been, has always gone hand in hand with science, which is what Researcher 1 was saying, and then not only that, but it has also always been concerned about the reality of the neighborhood, because it is a center for the neighborhood and the people of the neighborhood. So these two things together meant that when, on a social level, the subject of gender violence was beginning, it was just beginning, to be a subject that was given a little importance, then the school, being linked to people who are in the scientific field, saw the need for it to be one of the pioneers, because in fact it is, a pioneer, in having its own protocol, when it was beginning to be obligatory in many places and nobody had it, so the adult school was a pioneer in taking it on. Now it is something that most entities should have.

(FG_W_R2)

According to Researcher 2, the decision to systematize the school’s actions for the prevention of and attention to gender violence into a protocol largely stemmed from the non-academic women’s deep concern for the victims. This is why the school consults them as experts, as Researcher 1 also pointed out, as they provide protocols from other institutions based on scientific evidence. This co-creation approach is grounded in the school’s commitment to implementing actions with social impact based on scientific evidence, to the victims, and to the eradication of this social scourge:

Because the women in the adult school are people who are very concerned about victims in general. So that’s what made the school decide that. So, they consulted us, as Researcher 1 has already explained. And then we passed on protocols to them, which Researcher 1 has already explained, protocols that already existed. So, the school decided to apply a protocol following scientific evidence, which is what Researcher 1 has already explained.

(FG_W_R2)

### 3.2. RQ2: How Is This Protocol Implemented?

#### 3.2.1. Inclusion of the “Other Women” in Dissemination

The inclusion of “other women”, the non-academic women participating in adult education, in the dissemination of the protocol was a key element for its recognition not only by everyone in the school but also by everyone in the neighborhood and in other schools and associations. Researcher 2 explains how once the protocol had been written, it was promulgated in different spaces, such as the women’s group at the school. This group of women meets once a month to discuss topics of interest that they themselves have previously chosen. The protocol was also revealed in debate spaces for women participating in adult education within the FACEPA (the Catalan acronym for the Federation of Cultural and Educational Associations of Participants in Adult Education), to which the school that created it belonged. Therefore, the protocol of this school was an example for other schools and associations in this federation to follow as an action to advance their entities as safe spaces free from gender violence:

I think the Women’s Group also talked about it, I don’t know if it was only about the school, but also about FACEPA [Federation of Cultural and Educational Associations of Adults] in general. So, there was a lot of talk about how to raise awareness (about the protocol) so that people would experience the school as a safe environment.

(FG_W_R2)

Moreover, Researcher 2 explains the perception that this school is the safest environment in the neighborhood, and that, with the aim of continuing to improve it, “other women” have proposed several actions to promote the visibility of the protocol. For example, looking at the actions implemented at other institutions internationally, they created posters with slogans that highlighted the school as a safe space free from gender violence. This visibility of the protocol facilitated its adoption in other contexts in the neighborhood, such as the annual street concerts held during the local festival:

It has always been the safest environment in the neighborhood, I have no doubt about that. But how to continue improving in that sense. And that made the school (from the “other women” mainly) more visible, bigger signs were made and it was explained, ‘violence is rejected here’. They were also very much in line with what is being done at the international level. So, they also started to tell people about it. And above all, it was like people assumed that it was a tool for prevention that made the school a safe space and this was then transferred to different environments, as has always happened in schools, that from there it was transferred to other environments. That was commented on.

(FG_W_R2)

Participant 3 was very involved in the school women’s group that had disseminated the protocol throughout the school and the neighborhood. In her account, she also emphasized how the protocol has been adopted in other neighborhood spaces. Specifically, she referred to the fact that during various activities within the framework of the local neighborhood festival, the organization has created what is known in Catalan as the “Punt Lila”, which, in English, means purple point, referring to the color that represents women. This is a small tent with a counter on the street where any woman who suffers any type of aggression or abuse or who has been a bystander to it can go to report it. There, the person will be advised on their options and accompanied in whatever decision they make: “And in the neighborhood festivities, well, the same, there is a point that they put, a purple point and also there, in case you see anything (LS_W_Pa3)”.

Educator 1 explained that in the event of a potential case of gender violence in this adult school, the coordinator activates the Anti-Harassment Committee. In each case, the committee meets and discusses the best course of action. When the decision is made to talk to the aggressor about the case of violence, if they do not commit to change and to not repeating the violent behavior, as well as in cases in which the aggressor is a repeat offender or the violent behavior is serious, the perpetrator is asked to leave the school:

And then the coordinator activates the Anti-Harassment Committee. This Committee meets and advises on what should be done. Often it is a matter of talking to the person and either they commit to changing their attitude and not doing it again or measures will have to be taken. In some cases, some people have been told that they could not return because they were repeat offenders or because what they had done was very serious.

(I_W_E1)

Participant 3 explained the protocol at the start-of-term assembly, emphasizing how important the initiatives carried out by the school have been for the women in the neighborhood in terms of preventing and addressing gender violence: “Here when the Assembly has been held, it has been explained (…) This school, I tell you, this has been… a marvel, a marvel for the women (LS_W_Pa3)”.

Educator 1 explained how the protocol is also presented at the weekly coordination meetings of the team of educators working at the school so that new members are aware of it. Regarding its dissemination among collaborators (those who volunteer in various classes and activities at the school), the protocol is presented during the monthly coordination meeting, also to ensure that new collaborators are informed:

We also take the opportunity to make all this known. And then as a team, in the weekly coordination, it is a topic that is also very important. At the level of collaborators, we are informed in the monthly coordination that we have, especially now at the beginning of the course. We have also dedicated some time more specifically to this topic, because it is often a fundamental issue, so that everyone is aware of it and knows what to do, because of course, many classes are given by voluntary collaborators.

(I_W_E1)

Educator 2 emphasized the importance of everyone knowing the protocol and creating an environment wherein any incident can be reported the same day, which helps to avoid the victim’s suffering and prevents them from leaving the school:

It is very important that these things are communicated the same day they happen. And it doesn’t matter if I’m not there, because you call me, but it’s something that’s important, because if it doesn’t happen, maybe that girl won’t come to class next day.

(I_W_E2)

#### 3.2.2. The Inclusion of the “Other Women” in the Implementation

In relation to the implementation of this protocol, a committee of non-academic women participants, educators, and collaborating researchers was created. The inclusion of non-academic women participants on the committee in the implementation process, in an egalitarian dialogue with the collaborating researchers and educators, allows for a better response to the reality of the social contexts of the situations that may arise. Participant 2, a member of the committee, explained how the committee includes not only professional women, referring to educators working in schools and research collaborators in gender violence prevention, but also women, such as herself, without academic qualifications:

In this Committee there are researchers, there are people who are in charge of the day-to-day organization (educators of the school) and there are people who are at the grassroots. I don’t mind saying that I am there. So, when we meet (the Committee) we all agree on what we do. I am in this group (in the committee that deals with cases) even though I am not a professional or a teacher.

(FG_W_Pa2)

Moreover, Participant 1 stated that the committee, which addresses cases of gender violence at this school, ensures respect between all the diverse people involved. She further explained the relevance of breaking one’s silence on any case and reporting it, for which the school has the following options: in-person reporting at the school in a secure room, by phone, or by email, ensuring the welfare of the victim:

There is a Committee in charge of all this. There are different cultures and ages. We must respect everything, teach respect for everything. We must report. There is the ‘Raconet’ and you can call the school or the phone. You can send an email and then all of this will be treated with the most care for the person who has been forced to do it; nobody must keep quiet. Nobody must keep quiet.

(LS_W_Pa1)

Participant 2 emphasized that when the protocol is made known so that everyone is aware that they can report a case and that a committee will handle it, it is also communicated that the entire process is confidential, respecting the anonymity and privacy of the person reporting the situation:

It is not a question of everyone knowing about it either. What is said to all the people who find themselves in this situation is that they should report it, in the sense of reporting it to the school, that they are suffering this violence to be able to act. That they should not keep quiet about it and that there is a committee that will always support them, that they should not feel ashamed (…) And this is made known both to those who give classes and to the participants, and what is said is that this is a group that will always give support, that it will remain strictly confidential.

(LS_W_Pa2)

Researcher 2 specified that, following this protocol, when a possible case of violence is detected, the person who coordinates the team of educators communicates it to Participant 2. This participant is a referent, as she is one of the non-academic women at the school who has been the most involved in gender violence prevention in the community. In some cases, the advice of researchers on gender violence prevention who collaborate with the school is sought. Then, the decision as to which actions to implement is always made jointly between the members of the committee, the majority of whom are non-academic women. The decision always aims to support and protect the victim:

When there is a case in the school and it is detected, then they (the coordinators of the educator’s team) talk to Participant 2, who is the person in charge in this case, and from there, it is the school that assesses the follow-up of the protocol. So sometimes they call us, then we talk, or we do it online and we decide a little bit how to take it on, it depends on the situation of the case, it is given, it is taken to one of the spaces of the school of decision or to another. But always with the objective of supporting the victim.

(FG_W_R2)

Participant 2 stated that this way of working, with co-creation between the non-academic women participants, educators, and collaborating researchers, contributes to a better response to possible cases of violence because each party contributes their experiences from their perspective, including the day-to-day knowledge of the participants and the scientific evidence. Through this combination, Participant 2 expressed that more clarity is provided on the best way to proceed in each case:

I think so because each one has a different experience. I have a more personal day-to-day experience, and they have scientific experience, based on a lot of scientific evidence that can contribute to a lot of things. They can give clarity to what is being raised, that sometimes what seems to be something that can be tackled in one way does not have to be tackled in that way, it must be done with scientific guidelines and measures.

(FG_W_Pa2)

Researcher 2 provided an example of the egalitarian dialogue that took place between Participant 2 and the collaborating researchers, Researchers 1 and Researcher 2, in a meeting to guide the decision on how to implement one of the protocol points in a case, specifically regarding who to inform once the case is detected in order to always protect the victim. The experience of Participant 2, not only as a neighborhood resident but also as a woman committed to feminist efforts in preventing gender violence, was crucial. She is not an academic woman; however, her knowledge aligns with the proposal made by the researchers: to inform the minimum number of people (educators, collaborators, and participants) who will help protect the victim, with the victim’s consent:

To add an example, we met (for one case) and then it was just as you explained (she refers to Participant 2). You explained it very well. It was Participant 2 that gave the really correct view. We said, well, that will depend on how best to secure the environment, how best to ensure that the victim will be safe. Then Participant 2 said, ‘well I am here, knowing the people, seeing how it is, I agree with what you think. We reached a very oriented agreement because of the knowledge that Participant 2 has. Participant 2 has throughout her trajectory, also as a feminist, not just as a neighborhood resident. Participant 2 decided, agreeing with us that it was the best criteria that she gave, based on the protocol that we had previously agreed on, which was based on evidence, but the concrete orientation and it was a good decision. (…) At the time when this situation is happening, then the protocol said, ‘first you have to protect the victim and then you have to inform’, in agreement. So, the criterion was, who should we inform, the whole school board, the whole school, or just some of the teaching staff? How do we do it so that the person who is suffering the specific situation is really safe and it doesn’t become something that everyone can comment? Based on this, the criterion of who was informed was raised by Participant 2.

(FG_W_R2)

Researcher 1 also highlighted that, with this example, the functioning of the committee reflects how egalitarian dialogue is implemented in this school. She further added that this committee has a longstanding tradition within the school of emphasizing evidence-based arguments, whether they come from the knowledge of the collaborating researchers, the educators, or the women participants. The agreement is based on the argument that everyone considers valid, regardless of the power position of the person who advanced it. Researcher 1 explained that it is through this co-creation approach that cases of potential violence in the school are addressed and resolved:

This Committee also reflects how egalitarian dialogue is implemented in the school. It is a co-creation among diverse members, in this case, with different backgrounds; for instance, although both Researcher 2 and I are from the University, we also seek information from other participants and technical experts. The Committee embodies a longstanding tradition in the school of considering the evidence that we, along with others, can provide. The best part is that, as in the example Researcher 2 described, this co-creation approach guides how to respond to or resolve such cases.

(FG_W_R1)

#### 3.2.3. Upstander Behavior: Support for the Victim from the Outset and Throughout the Entire Process

In the process of implementing and using this protocol, a fundamental aspect is the support provided to the victim from the very beginning and throughout the entire process. One of the female educators (Educator E3) of this school voluntarily offered to explain her case as an example of success when she heard that a qualitative case study was being conducted on the process of creating and implementing this protocol. She believes that the care with which the committee handled her case and the support she received from the very beginning, both from the committee and from the members of the educators’ team who were informed, with her consent, made her feel always protected. She wanted to highlight that the suffering she endured due to the harassment by a participant of the school was transformed into a feeling of calm upon receiving support and the adoption of measures. Among these measures, the coordinator of the school and a member of the committee (E1) warned the person who had harassed the female educator that if his behavior did not change, he would not be allowed to return to the school. In addition, she (E1) made him delete some videos he took of the educator (E3) without her consent on a field trip outside the school from his mobile phone. The educator (E3) was protected so that she would not be alone in a space where this person was present; nevertheless, as the harassing behavior did not change, the person was not allowed to access the school. Educator 3 explained it as follows:

The feeling of protection from all sides as far as the school was concerned was spectacular, from the fact that they made him delete the videos from his mobile phone, to the fact that the coordinator came on Sunday, to the fact that they changed my shift, that he was no longer allowed to enter the civic center or the school, to this person, that is to say, the truth is that the anguish that it generated in me, on the one hand, gave me calm on the other hand.

(LS_W_E3)

The team of educators who were informed made the educator feel protected. She felt grateful because her colleagues were attentive, especially in the first few days. They let her know that she could ask for their help at any time, for example, when she was leaving the school to go home:

The truth is that I was very grateful to the team because I felt very well supported. I felt very protected and in fact, in the first few days they also told me that if I went out and noticed that I was close to such and such, to call and run or to go back to school.

(LS_W_E3)

In this case, the educator decided to report the harassment to the police, a decision that was fully supported by the school’s committee.

## 4. Discussion

One of the characteristics highlighted in this study in response to the first research question concerning the creation of a protocol for the prevention of gender violence in a non-university adult educational center is the ongoing commitment to preventing gender violence and supporting victims. The systematization of this protocol was a formalization of the processes and accumulated experience that the school had implemented since its inception. The feminist approach that is maintained at the school to identify and address the needs of all women fosters a safe emotional environment within the school by promoting attitudes and behaviors that ensure security, freedom of choice, and solidarity among all. These findings align with other research that demonstrates the benefits of supportive actions in protecting against the psychological consequences for victims of gender violence (Erčulj & Pavšič Mrevlje, 2023; Gao et al., 2024) and those who support them (Flecha et al., 2024; Aubert & Flecha, 2021). This is in line with previous research that points out the need to address IGV (Vidu et al., 2021) and protect those who support the prevention of gender violence and its health consequences, as well as to reduce mental health issues in the context of student violence against supporters (Gregory et al., 2017; Perry et al., 2024).

A key milestone of this protocol is that it has been co-created, involving women participants without higher education degrees, researchers, and educators from the school. In the process of co-creating and implementing this protocol, “other women” (Puigvert, 2001) have contributed to preventing gender violence at this adult school, as reported by the participants. The “other women” who participate in adult education and have experienced struggles in the neighborhood and villages on the basis of popular education projects suffer double or triple discrimination for their lack of access to education and some of them also for being immigrants (Nic a Bháird, 2013; García Yeste et al., 2011) or belonging to cultural minorities such as the Roma community (Beck-Gernsheim et al., 2003). When participants of this adult school decided to systematize the measures they were already conducting into a protocol, they did not find any evidence of previous implementation of such a protocol at any non-university educational centers. The participants pointed out that this protocol was agreed upon through egalitarian dialogue, based on the school’s prior experience and scientific evidence that had previously shown the results of preventing violence in other educational contexts (Gatt et al., 2011; Villarejo-Carballido et al., 2019). Along these lines, further research has underlined the benefits of involving “other women” in the co-creation of processes for achieving spaces free from violent behaviors (Oliver et al., 2009) and improving the mental health and well-being of those involved (Padrós-Cuxart et al., 2024).

Addressing the second research question regarding the implementation of this protocol, a key psychosocial factor identified in this study is the role of the “other women” in disseminating the actions outlined in the protocol across various contexts, both within and beyond the school environment. This dissemination process has been instrumental in fostering psychologically safe spaces, wherein individuals feel empowered to report incidents of violent behavior. These findings are consistent with prior research indicating that the involvement of “other women” in the application of interventions targeting gender violence enhances the creation of violence-free communities (Valls Carol, 2014). Such involvement has been shown to cultivate prosocial attitudes, including solidarity (Pulido et al., 2014), and to promote positive social interactions that improve interpersonal dynamics and overall coexistence (Serradell et al., 2019). A salient intervention implemented within this protocol was the establishment of a committee comprising non-academic female participants alongside educators and collaborators. This group includes volunteers from diverse educational and social backgrounds, ranging from women who recently achieved literacy to doctoral researchers affiliated with the university. The committee’s role is to address cases of gender violence in this school’s setting while maintaining respect among all participants and upholding strict confidentiality for victims throughout the reporting and resolution process by ensuring that they feel psychologically safe and supported when reporting (Acurio et al., 2023).

The unified commitment of the school community to the adoption of a victim-centered approach has been a cornerstone of this protocol, significantly contributing to the prevention of gender violence. Immediate and coordinated actions to support victims have established a clear and consistent stance that fosters upstander behavior, active intervention to prevent or address violent acts. Previous research has underscored the importance of engaging witnesses of violence as a strategy to reduce both victimization and perpetration in educational contexts (Coker et al., 2016). Upstander behavior, which involves acting to stop or prevent violence rather than passively observing it, plays a critical role in this process (Villarejo-Carballido et al., 2019). The creation of safe and empowering conditions for individuals to voice concerns and intervene has been identified as essential for preventing violent behaviors (Puigvert et al., 2022). Participants of this school recognize this protocol as instrumental in fostering such conditions, not only ensuring the safety of victims but also providing a secure environment for upstanders to act confidently against violence.

The limitations of this study stem from its focus on a specific case with particular characteristics. The processes and characteristics analyzed in this study are specific to this case, and this must be taken into account when interpreting and generalizing the results. Furthermore, this study is limited to the creation and implementation processes of this protocol. Nonetheless, this specificity allows for the advancement of scientific knowledge and the development of future lines of research that address aspects not analyzed in this study.

## 5. Conclusions

This study presented a case demonstrating the successful development and implementation of a protocol for addressing gender violence in a non-university adult education center with the active involvement of the community, particularly “other women” (Puigvert, 2001). The findings highlight the critical characteristics of this protocol, emphasizing the inclusion of diverse voices in its co-creation, including women without higher education degrees. Furthermore, the protocol’s foundation in scientifically supported evidence with proven social impact has been integral to its success in preventing gender violence in educational settings.

A central aspect of this protocol is its focus on addressing IGV (Flecha et al., 2024), promoting actions that protect both victims and their supporters. This focus has been identified as key to enhancing the psychological well-being of all parties involved, which is essential for the protocol’s effectiveness. These insights offer valuable contributions for future research and the development of similar protocols and interventions, particularly in adult education centers and other environments where measures against violence may not yet be implemented.

This study underscores the importance of co-creation and implementation processes, which foster violence-free spaces and, consequently, promote psychological well-being. The protocol’s design and execution address broader public health and mental health needs regarding the prevention of gender violence. By encouraging upstander behaviors—through immediate support and advocacy for victims—the protocol serves to create a supportive environment that mitigates the harmful psychological effects of violence. These findings contribute to a deeper understanding of effective strategies for the prevention of violence, and offer a valuable framework for future interventions in various educational and social contexts.

## Figures and Tables

**Table 1:** Participant descriptions.

Code	Profile	Data Collection	Gender	Age Range	ISCED *
E1	Educator	Interview	Woman	30–35	L5
E2	Educator	Interview	Woman	45–50	L6
E3	Educator	Life Story	Woman	30–35	L5
Pa1	Participant	Life Story	Woman	80–85	L0
Pa2	Participant	Life Story, Focus Group	Woman	75–80	L1
Pa3	Participant	Life Story	Woman	70–75	L1
R1	Researcher	Focus Group	Woman	65–70	L6
R2	Researcher	Focus Group	Woman	50–55	L6

* International Standard Classification of Education (UNESCO Institute for Statistics, 2012). NBS: no basic studies; L0: preprimary education; L1: primary education 1–6; L2: lower secondary education 1–4; L3: upper secondary education 1–2; L4: postsecondary nontertiary education; L5: first stage of tertiary education 1–3/4; L6: second stage of tertiary education ½.

## Data Availability

The datasets are not directly available online to ensure the necessary level of confidentiality and the legitimate utilization of the data. Researchers interested in accessing any of the datasets are kindly asked to make a formal request by sending an email to Rosa Valls-Carol. This request should be accompanied by the following documents: a formal letter containing the researcher’s contact information, institutional affiliation, current position, the purpose of the research, details regarding the intended use of the data and, if applicable, information about funding sources; an official letter from the researcher’s affiliated university or research institution confirming their association; and a confidentiality agreement, duly signed by the researcher, indicating their commitment to maintaining the confidentiality of the data.
